# Correlating Biomechanical Gait Analysis With Patient-Reported Outcomes After Hip Arthroscopy for Femoroacetabular Impingement Syndrome

**DOI:** 10.1177/23259671221121352

**Published:** 2022-09-05

**Authors:** Abhishek S. Kannan, Matthew J. Hartwell, Trevor Grace, Eric Hammond, Kylen K.J. Soriano, Richard B. Souza, Alan L. Zhang

**Affiliations:** * Department of Orthopaedic Surgery, University of California, San Francisco, San Francisco, California, USA.; † Musculoskeletal and Quantitative Imaging Research Group, Department of Radiology and Biomedical Imaging, University of California, San Francisco, San Francisco, California, USA.; *Investigation performed at the Department of Orthopaedic Surgery, University of California, San Francisco, San Francisco, California, USA*

**Keywords:** biomechanics, femoroacetabular impingement, gait analysis, hip arthroscopy, outcome

## Abstract

**Background:**

Postoperative biomechanics after hip arthroscopy for femoroacetabular impingement syndrome (FAIS) are an outcome of interest, but correlation with patient-reported outcomes (PROs) remains unclear.

**Purpose/Hypothesis:**

The purpose of this study was to assess the correlation between changes in hip biomechanics in FAIS patients after hip arthroscopy and changes in PRO scores. We hypothesized that gait analysis would demonstrate significant correlations between pre- and postoperative changes in biomechanics and changes in PRO scores.

**Study Design:**

Descriptive laboratory study.

**Methods:**

FAIS patients without dysplasia or arthritis who underwent primary hip arthroscopy for labral repair and femoroplasty underwent preoperative and 1-year postoperative 3-dimensional motion tracking and biomechanical testing during normal gait. Joint kinematics calculated included flexion/extension (sagittal plane), abduction/adduction (frontal plane), and internal/external rotation (transverse plane). Peak hip angles and moments were compared between baseline and 1-year postoperative measures. At baseline, 1-year, and 2-year postoperatively, patients completed the following PRO surveys: 12-Item Short Form Health Survey (SF-12), modified Harris Hip Score (mHHS), and Hip disability and Osteoarthritis Outcome Score (HOOS). Joint kinematics that significantly improved 1 year after surgery were assessed for correlations with PRO scores.

**Results:**

A total of 10 patients (12 hips) were enrolled prospectively. PROs significantly improved at 1 and 2 years postoperatively compared with baseline values for HOOS, mHHS, and SF-12 Physical Component Score, with all patients achieving the minimal clinically important difference (MCID) on the HOOS Sport/Recreation and Quality of Life subscales. From preoperatively to 1-year postoperatively, significant improvements were seen in peak hip abduction angle (from −2.3° ± 1.8° to −4.6° ± 1.8°; *P* = .0058) and peak hip extension moment (from −1.03 ± 0.19 to −0.85 ± 0.20 N·m/kg; *P* = .014); however, there were no significant correlations between these changes and the pre- to postoperative changes on any PRO scores.

**Conclusion:**

Gait analysis of FAIS patients after hip arthroscopy demonstrated small, albeit significant, changes in postoperative hip kinetics and kinematics; however, these changes did not correlate with the large, clinically significant improvements in PROs at 1 year after surgery.

**Clinical Relevance:**

The results of this study suggest that the degree of improvement in short-term PROs after hip arthroscopy for FAIS may not be related to small changes in biomechanics postoperatively.

Hip arthroscopy has become the standard of care in surgical management of femoroacetabular impingement (FAI) syndrome (FAIS) and serves as a minimally invasive, less morbid, effective means of addressing bony deformity and chondrolabral pathology. An increasing rate of hip arthroscopy utilization has been demonstrated in the literature, showing a 365% increase from 2004 to 2009.^30^ A more recent study confirmed the persistence of this trend, with 250% more procedures performed over a 4-year span from 2007 to 2011.^43^ Contemporary literature has demonstrated a high rate of return to sport,^9,12,27,28^ as well as significant improvements in pain, function, and patient satisfaction at short-,^2,11,26,37^ mid-,^16,34^ and long-term follow-up.^23^ Studies evaluating postoperative biomechanical outcomes, however, have remained sparse. Several studies in the past decade have introduced functional testing and motion analysis as a potential outcome of interest after hip arthroscopy. However, findings remain inconsistent, with varying motion analysis protocols and unclear correlation with patient-reported outcomes (PROs).

Previous research has provided data to support altered hip kinematics when comparing FAIS patients with healthy controls.^1,15,20,24^ However, few studies have evaluated postoperative changes in gait biomechanics after hip arthroscopy.^4,10,38,39^ Rylander et al^38^ studied hip and pelvic kinematics during walking and stair climbing in 17 FAIS patients and noted that, at 1 year postoperatively, abnormal kinematics were restored to normal in the operative limb during walking (statistically significant increases in maximum hip flexion, rotation range of motion, and maximum internal rotation) but not during stair climbing. More recently, Cvetanovich et al^10^ evaluated double-leg squat and gait biomechanics in 15 FAIS patients at 6 months after hip arthroscopy, demonstrating a significant decrease in peak external hip extension moment during a double-leg squat postoperatively compared with preoperative baseline. However, the authors found no significant differences when comparing other measurements during double-leg squat or gait biomechanics.^10^ There remain no studies to date that have aimed to correlate postoperative change in biomechanics with PROs.

The purpose of this study was to assess changes in hip joint biomechanics in FAIS patients treated with hip arthroscopy and to correlate changes with changes in PRO scores. We hypothesized that there would be significant correlations between biomechanical gait analysis and PRO score changes in FAIS patients by 1 year after hip arthroscopy.

## Methods

### Patient Selection and Data Collection

Institutional review board approval for the study protocol was obtained from our institution, and patients were enrolled prospectively after giving written informed consent. A single sports medicine fellowship-trained surgeon with a focus on hip arthroscopy (A.L.Z.) performed all surgical procedures in this study. Inclusion criteria consisted of patients diagnosed with FAIS indicated for hip arthroscopy who had cam-type FAI and labral tear with failure of nonoperative treatment, including activity modification and physical therapy of 3-month duration. An alpha angle ≥55° was used to indicate cam impingement. Exclusion criteria included hip dysplasia (lateral center-edge angle [LCEA] <25°), osteoarthritis (Tönnis grade >1), and hypermobility (Beighton score ≥4). Intra-articular injections before arthroscopy were used for diagnostic and therapeutic purposes but were not a strict inclusion criterion, as some patients refused injections and elected for surgical treatment after physical therapy and nonoperative treatment failed.

Preoperative baseline kinematics data and surveys were collected before surgery, and postoperative kinematic and PRO data were analyzed at 1 year; 2-year postoperative PROs were also collected. Patient demographics, such as age, sex, and body mass index (BMI), were recorded. Patients underwent radiographic evaluation, which included pre- and postoperative radiographs of the pelvis in the supine anteroposterior plane and Dunn lateral 45° views, as well as preoperative magnetic resonance imaging of the affected hip.^36^ Radiographic measurements, including alpha angle, LCEA, and Tönnis grade, were recorded.

### Surgical Treatment and Rehabilitation

All procedures were performed in the ambulatory surgery center of a tertiary referral academic medical center. Two arthroscopic portals (anterolateral and midanterior) were utilized. Acetabular, femoral, and labral condition was recorded per the classification by Beck et al.^3^ All patients underwent arthroscopic labral repair and femoroplasty through a periportal capsulotomy without closure.^8,29^ No concomitant procedures such as psoas tenotomy were performed. Postoperatively, all patients were limited to touch-down weightbearing with crutch use for 2 weeks. After 2 weeks, patients were allowed to advance weightbearing as tolerated. A comprehensive physical therapy regimen was utilized for rehabilitation and strengthening, with progression to a running program at 3 months after surgery and return to sports at 5 to 6 months after surgery.

### Patient-Reported Outcomes

Patients completed 3 PRO surveys pre- and postoperatively: the 12-Item Short Form Health survey (SF-12), modified Harris Hip Score (mHHS), and Hip disability and Osteoarthritis Outcome Score (HOOS). These PROs were validated in past studies of hip arthroscopy outcomes to assess a patient’s pain, functional status, and quality of life (QoL).^19,40^ The SF-12 survey contains a Physical Component Summary (PCS) and a Mental Component Summary (MCS) to assess general health-related QoL.^13,17,18,47^ The mHHS survey produces a single score assessing hip function.^6,14,17^ The HOOS provides 5 subsection scores: Symptoms, Pain, Activities of Daily Living (ADL), Sport/Recreation, and QoL.^31,32^ In addition, patients rated their pain pre- and postoperatively on a visual analog scale (VAS) from 0 to 10, with 0 referring to no pain and 10 referring to the most pain. All data were collected in REDCap (Version 8.1.4).

### Motion Analysis Data Acquisition

Three-dimensional (3D) motion tracking and biomechanical testing was performed using previously validated methods developed by the University of California, San Francisco, for review. Marker trajectory data were collected using a 10-camera motion-analysis system (VICON: Oxford Metrics) set at 250 Hz while ground reaction forces (GRF) were sampled simultaneously at 1000 Hz using 2 inground force plates (AMTI). A marker set of 45 retroreflective markers was attached to each participant to create the rigid body segments necessary to capture kinematics. Calibration markers were placed on the head of the first metatarsal, medial and lateral malleoli, medial and lateral femoral epicondyles, and the greater trochanter of both left and right lower limbs. Rigid body clusters consisting of 4 markers were placed on the lateral sides of the thigh and shank, and rigid body clusters consisting of 3 markers were placed on the heel shoe counter. Additional tracking markers were attached on left and right acromion, C7 vertebrae, sternal notch, L5/S1 joint, anterior superior iliac spines, iliac crests, and head of the fifth metatarsal. Participants wore nonrestrictive clothing and the same shoe type (running shoe, model 880; New Balance) to reduce the impact of shoe type on natural gait.

Marker trajectory and GRF data were both low-pass filtered with a fourth-order Butterworth filter with cutoff frequencies at 6 Hz and 50 Hz using Visual3D (C-Motion). A musculoskeletal model consisting of 8 segments was created for each participant in Visual3D from his or her respective standing calibration trial. The pelvis and thorax segments were modeled as cylinders while the thigh, shank, and foot segments were modeled as frusta of cones. This motion analysis acquisition has been utilized and validated in previous studies.^22,25,42,46^

### Motion Analysis Protocol

After performing a 1-second static calibration trial, all calibration markers were then removed. Each participant was asked to perform a total of 10 successful fixed speed walking trials (5 trials bilaterally at 1.35 m/s verified through timing gates). This speed was chosen because it is the average of the group means of men and women during normal free gait on a smooth surface.^35^ Participants were allowed to practice to become familiar with the protocol. A successful trial was defined as walking within the walking speed window (1.35 ± 0.07 m/s) as well as having the tested limb falling completely within the borders of the force plate.

### Motion Analysis Data Processing and Analysis

Gait kinetics and kinematics were observed during the stance phase of the limb in contact with the force plate and were time normalized to 101 data points. Initial contact during the stance phase was defined as when the foot applied a vertical GRF >20 N, and toe-off was defined as a vertical GRF between foot and force plate <20 N. External joint moments were derived from standard inverse dynamics equations and normalized by body mass within Visual3D. A local orthogonal coordinate system of the model segments was derived from the standing calibration trial. Segment position and orientation was estimated using an unweighted least squares optimization.^45^ Joint kinematics were calculated using a Cardan rotation sequence in the following order: flexion/extension (sagittal plane), abduction/adduction (frontal plane), and internal/external rotation (transverse plane). Variables of interest include peak ankle, knee, and hip angles and moments in the sagittal, frontal, and transverse planes, and were compared between baseline and 1-year measures. Peak angle and moments were found by averaging the sum of the peaks of 5 independent trials.

### Statistical Analysis

Postoperative PROs were compared with preoperative PROs using paired Student *t* tests. The percentage of patients achieving the minimum clinically important difference (MCID) was then calculated. MCIDs were defined for each PRO instrument using the distribution-based method as a score greater than half of the standard deviation from the mean of the preoperative score.^33^ Peak joint angles and moments were compared from baseline to 1 year after surgery using paired Student *t* tests. For joint angles and kinematics that improved significantly 1 year after surgery, we then assessed for correlations between joint kinematics and PRO scores. This was done by calculating the correlation coefficients between pre- to postoperative changes in PRO scores and pre- to postoperative changes in joint kinematics. *P* < .05 was considered statistically significant for all calculations. All statistical analyses were performed in Excel for Mac (Version 16.49; Microsoft).

An a priori power analysis determined that a study population of 9 patients would provide 80% power to detect a 25% change in peak joint moments for hip flexion.^41^

## Results

### Patient Characteristics

The study evaluated 12 hips from 10 patients, with a mean age of 32.4 years and mean BMI of 23.6. There were 6 female hips and 6 male hips, and patients had an average preoperative alpha angle of 63.7° and LCEA of 32.6°. Patient demographics can be found in Table 1.

**Table 1:** Patient Demographic Characteristics (N = 10 Patients, 12 Hips)*^a^*

Variable	Value
Age, y	32.4 ± 4.9
BMI	23.6 ± 3.3
Sex	
Male	50.0 (6)
Female	50.0 (6)
Side	
Left	41.7 (5)
Right	58.3 (7)
Alpha angle, deg	63.7 ± 5.9
LCEA, deg	32.6 ± 5.1

*^a^* Data are reported as mean ± SD or % (No. of patients). BMI, body mass index; LCEA, lateral center-edge angle.

### Patient-Reported Outcomes

PROs improved significantly at 1 and 2 years postoperatively compared with baseline values for all PRO instruments, except for the SF-12 MCS and pain VAS (Table 2). There were no significant changes in PRO scores from 1 to 2 years postoperatively for any of the PRO instruments. The percentage of patients achieving MCID at 1 year after surgery can be found in Table 3. All of the patients achieved MCID for the Sport/Recreation and QoL subscales of the HOOS and the MCS of the SF-12 had the smallest portion achieving MCID at 25.0%.

**Table 2:** Preoperative, 1-Year, and 2-Year Postoperative Mean PRO Scores*^a^*

				*P*		
PRO	Preop	1-y Postop	2-y Postop	Preop vs 1-y Postop	Preop vs 2-y Postop	1- vs 2-y Postop
mHHS	72.7 ± 9.4	87.5 ± 6.4	88.6 ± 12.3	**<.001**	**.0051**	.78
HOOS						
Symptoms	59.2 ± 9.5	74.2 ± 16.5	72.9 ± 13.6	**.030**	**.010**	.79
Pain	60.4 ± 12.3	81.3 ± 16.4	84.2 ± 16.1	**.0034**	**.0017**	.56
ADL	69.1 ±15.6	87.4 ± 18.6	90.3 ± 11.6	**.013**	**.0012**	.39
Sport/Recreation	39.1 ± 14.4	78.7 ± 17.2	75.0 ± 25.4	**<.001**	**<.001**	.47
QoL	25.5 ± 12.0	60.4 ± 15.6	62.5 ± 21.8	**<.001**	**<.001**	.70
SF-12						
PCS	32.3 ± 8.0	48.4 ± 9.4	47.3 ± 11.9	**<.001**	**.0040**	.80
MCS	39.3 ± 11.0	41.6 ± 14.1	38.9 ± 15.0	.50	.93	.26
VAS pain	3.3 ± 2.0	2.2 ± 1.9	2.3 ± 2.0	.19	.17	.91

*^a^* Data are reported as mean ± SD. Boldface *P* values indicate statistically significant difference (*P* < .05; paired *t* test). ADL, Activities of Daily Living; HOOS, Hip disability and Osteoarthritis Outcome Score; MCS, Mental Component Summary; mHHS, modified Harris Hip Score; PCS, Physical Component Summary; PRO, patient-reported outcome; QoL, Quality of Life; SF-12, 12-Item Short Form Health Survey; VAS, visual analog scale; Preop, preoperative; Postop, postoperative.

**Table 3:** Patients Reaching MCID at 1 Year After Hip Arthroscopy*^a^*

PRO	MCID Value*^b^*	Achieving MCID, %
mHHS	4.7	91.7
HOOS		
Symptoms	4.8	58.3
Pain	6.7	83.3
ADL	7.8	75.0
Sport/Recreation	7.2	100.0
QoL	6.0	100.0
SF-12		
PCS	4.0	83.3
MCS	5.5	25.0
VAS pain	1.0	75.0

*^a^* ADL, Activities of Daily Living; HOOS, Hip disability and Osteoarthritis Outcome Score; MCID, minimal clinically important difference; MCS, Mental Component Summary; mHHS, modified Harris Hip Score; PCS, Physical Component Summary; PRO, patient-reported outcome; QoL, Quality of Life; SF-12, 12-Item Short Form Health Survey; VAS, visual analog scale.

*^b^* Calculated using the distribution-based method.

### Joint Kinematics

**Figure 1.**
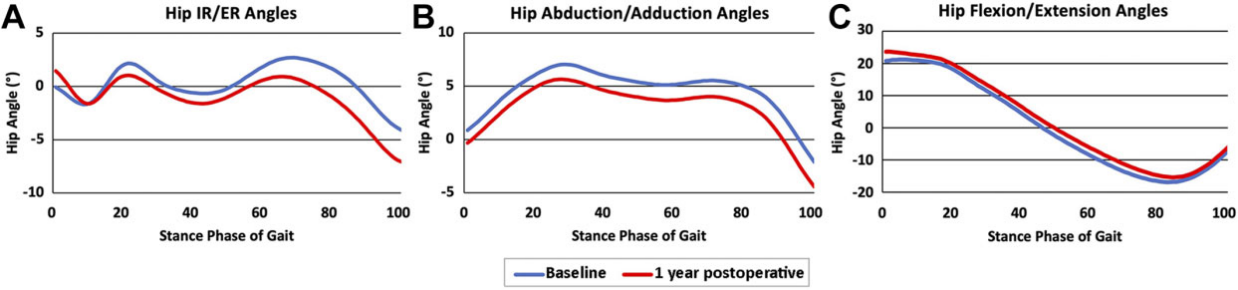
Peak hip joint angles in the (A) sagittal, (B) frontal, and (C) transverse planes during the stance phase of gait at baseline and 1 year after hip arthroscopy. ER, external rotation; IR, internal rotation.

**Figure 2.**
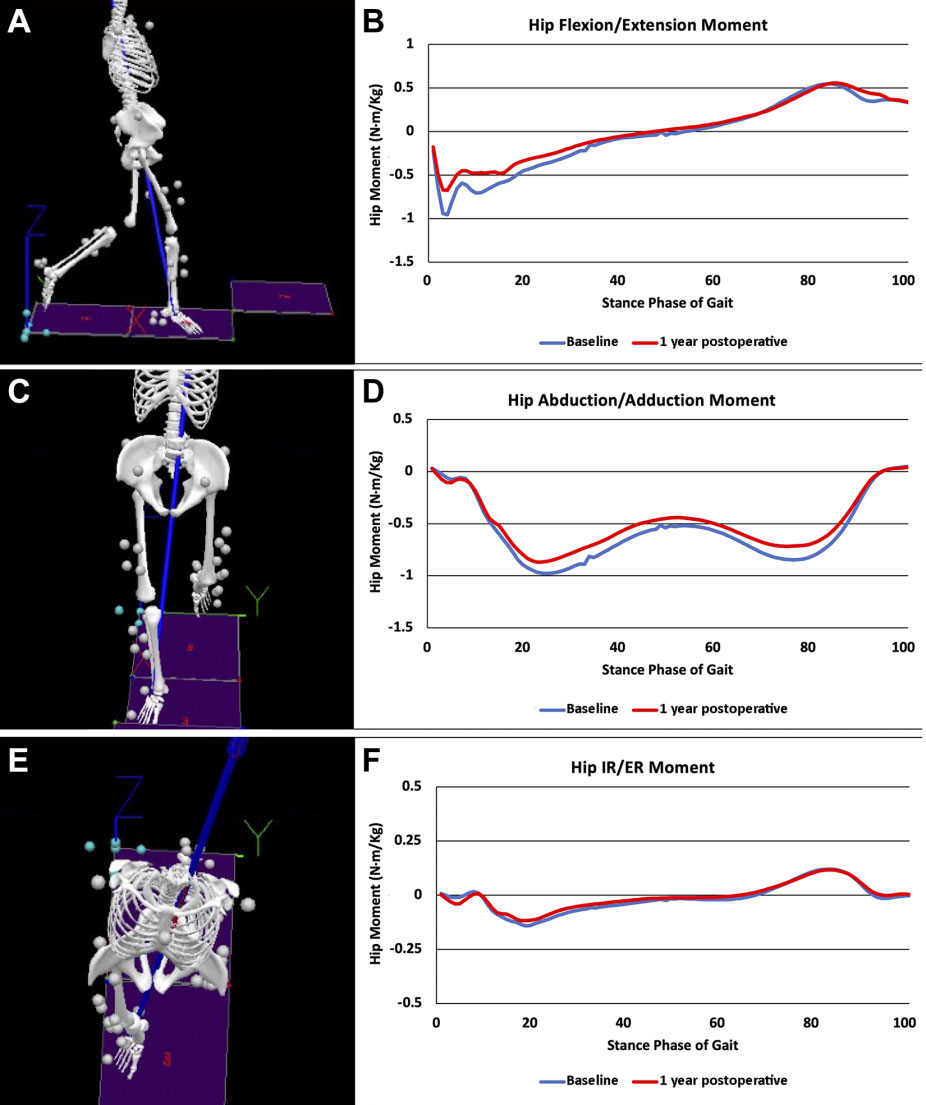
(A, C, E) Marker position data tracked with a motion capture system and used to create an 8-segment musculoskeletal model to calculate lower extremity joint moments during walking. (B, D, F) Peak hip joint moments in the (B) sagittal, (D) coronal, and (F) transverse planes during the stance phase of gait for femoroacetabular impingement syndrome patients at 1 year after hip arthroscopy. ER, external rotation; IR, internal rotation.

Hip abduction was the only peak joint angle that changed at 1 year postoperatively, increasing from −2.3° ± 1.8° to −4.6 ± 1.8° (*P* = .0058). Hip extension was the only peak joint moment that was significantly changed at 1 year postoperatively, reducing from −1.03 ± 0.19 to −0.85 ± 0.20 N·m/kg (*P* = .014). The remaining joint kinematic analyses can be seen in Table 4. Graphical representations of the average joint angles (Figure 1) and moments for baseline and 1-year postoperative values throughout the phase of gait can be seen in Figure 2A to F.

**Table 4:** Comparison of hip Joint Kinematic and Kinetic Variables During Gait Between Baseline and 1 Year Postoperatively*^a^*

	Baseline	1-y Postop	*P*
Peak joint angle, deg			
Flexion	21.5 ± 7.9	24.0 ± 5.9	.30
Extension	−17.0 ± 7.1	−15.5 ± 6.4	.27
Adduction	7.8 ± 2.5	6.4 ± 2.5	.062
Abduction	−2.3 ± 1.8	−4.6 ± 1.8	**.0058**
IR	5.5 ± 5.8	4.6 ± 5.8	.62
ER	−5.5 ± 6.5	−8.9 ± 3.4	.0504
Peak joint moment, N·m/kg			
Flexion	0.59 ± 0.16	0.66 ± 0.13	.32
Extension	−1.03 ± 0.19	−0.85 ± 0.20	**.014**
Adduction	0.10 ± 0.05	0.08 ± 0.05	.43
Abduction	−1.04 ± 0.21	−1.00 ± 0.11	.46
IR	0.13 ± 0.07	0.14 ± 0.08	.92
ER	−0.17 ± 0.08	−0.16 ± 0.06	.84

*^a^* Boldface *P* values indicate statistically significant difference (*P* < .05, paired *t* test). ER, external rotation; IR, internal rotation; Postop, postoperatively.

### Correlation Between Joint Kinematics and PROs

Correlation coefficients between joint kinematics and PRO correlations can be found in Table 5. There were no significant correlations between the pre- to postoperative changes in hip abduction angles/extension moments and the pre- to postoperative changes in PRO scores.

**Table 5:** Correlation Between Pre- and Postoperative Kinematic Changes and PRO Changes*^a^*

	Δ_PRO_								
Δ_Kinematics_	mHHS	HOOS–Symptoms	HOOS–Pain	HOOS-ADL	HOOS–Sport/Recreation	HOOS-QoL	SF-12 PCS	SF-12 MCS	VAS Pain
Abduction angle									
*r*	0.02	−0.02	−0.20	−0.39	−0.01	0.05	−0.38	0.12	0.04
*P*	.95	.94	.53	.22	.97	.87	.22	.72	.91
Extension moment									
*r*	−0.18	−0.18	−0.15	−0.32	−0.25	−0.14	−0.43	−0.16	0.13
*P*	.58	.57	.65	.32	.43	.66	.17	.63	.70

*^a^* ADL, Activities of Daily Living; HOOS, Hip disability and Osteoarthritis Outcome Score; MCS, Mental Component Summary; mHHS, modified Harris Hip Score; PCS, Physical Component Summary; PRO, patient-reported outcome; SF-12, 12-Item Short Form Health Survey; QoL, Quality of Life; VAS, visual analog scale.

## Discussion

The primary findings of this study include increased peak hip abduction angle and reduced peak hip extension moment during gait at 1 year postoperatively compared with preoperative baseline for FAIS patients undergoing hip arthroscopy. There were no other differences in hip joint kinetics and kinematics when comparing preoperative with postoperative measurements. With respect to PROs, patients reported significant improvements in nearly all outcome measures up to 2 years postoperatively, with a high percentage of patients achieving the MCID, which is consistent with recent literature on postarthroscopy outcomes.^11,23,28,37^ Finally, while the current results indicate that hip joint kinetics and kinematics change during gait 1 year after hip arthroscopy, these changes were not significantly correlated with PRO measures.

Our findings are consistent with results from Cvetanovich et al,^10^ who evaluated squat and gait biomechanics in 15 FAIS patients at baseline and 6 months postoperatively, and compared the surgical cohort with a healthy control group. Their results for double-leg squat showed that FAIS patients demonstrated a greater preoperative peak external hip extension moment during squat when compared with 6 months postoperatively, but there were no significant differences in any biomechanical variables during gait when comparing preoperative with 6-month postoperative FAIS patients. Compared with controls, the FAIS group at 6 months postoperatively demonstrated slower hip flexion joint angular velocity during the descent and ascent phases of squat, but showed no significant differences in joint kinetics or kinematics. There were also no significant biomechanical differences noted during gait between these 2 groups. The authors noted a significant improvement in Hip Outcome Score (HOS) Sports-Specific Subscale and HOS-ADL subscales postoperatively compared with preoperatively. Our current study demonstrated a similar significant decrease in peak external hip range of motion in postoperative patients during normal gait at 1 year postoperatively. We also saw a significant improvement in PROs in our cohort, and further correlation analysis revealed no relationship between PRO changes and kinematic changes after surgery.

Previous kinematic studies have also evaluated hip joint biomechanics during stair climbing.^38^ Rylander et al^38^ evaluated postoperative kinematics during walking and stair climbing in 17 patients and noted significantly increased sagittal plane range of motion when comparing postoperative FAIS patients with controls. The authors noted a significant increase in maximum hip flexion and internal rotation from pre- to postoperative during walking, contrasting with the current study findings of insignificant change in both flexion and internal rotation. While the authors reported significant differences in sagittal plane range of motion, hip extension, and rotational profile when comparing postoperative FAIS patients with controls during stair climbing, there were no significant changes in postoperative hip kinematics when compared with preoperative baseline within the surgical group.^38^

Whereas Cvetanovich et al^10^ did not include peak abduction angle in their kinematic evaluation, Rylander et al^38^ did not note a significant difference in this parameter between patients either pre- and postoperatively during walking or stair climbing. The findings of the current study contrast with those of Rylander et al,^38^ demonstrating a significant increase in postoperative hip abduction angle. Kubiak-Langer et al^21^ used a validated 3-D computed tomography-based kinematic analysis and noted that hips with FAI had decreased flexion, internal rotation, and abduction. While we found no significant increases in postoperative peak hip flexion or internal rotation, a significant increase in peak joint abduction angle was noted in the current study. Finally, the lack of pre- to postoperative change in most hip joint kinematics seen in our study is consistent with the findings of Brisson et al,^5^ although the latter evaluated patients treated via open or combined approaches necessitating surgical hip dislocation for cam resection. The study involved 10 cam-predominant FAIS patients evaluated within a range of 10 to 32 months postoperatively and found no significant differences between preoperative and postoperative groups regarding all kinematic and kinetic parameters.

PROs improved at both 1- and 2-year postoperative timepoints for this patient cohort, but improvement did not correlate with changes in hip joint kinetic or kinematic parameters. This may be explained partly by the fact that motion analysis of hip joint kinematics was limited to level walking. Although FAIS may be more clinically evident during activities requiring end range of motion such as hip flexion and internal rotation, our previous analyses have demonstrated that kinematic changes during normal gait can be correlated with preoperative FAI symptoms as well as intra-articular cartilage injury.^41,42^ Therefore, we found it valuable to analyze the pre- to postoperative changes for walking in this study. However, our initial hypothesis that kinematic changes in walking would correlate with PRO improvement was not supported by these data, as relatively small differences in biomechanical parameters from pre- to postoperative evaluation did not correlate with large improvements across different PROs. It is possible that PRO improvements are better correlated with biomechanical changes in higher level activities and sports, which is a goal for future research. It is also worth mentioning that patient demographics in this study reflect multiple factors associated with improved outcomes after hip arthroscopy, including younger age, lower BMI, and Tönnis grade <1.^7,44^ Thus, patient selection, adequate surgical correction, and postoperative rehabilitation remain crucial to achieving excellent outcomes after hip arthroscopy for surgical correction of FAIS, and these outcomes may likely be independent of subsequent, subtle change in joint mechanics for low-level activities such as walking.

### Limitations

This study is subject to limitations. There is limited generalizability of the findings due to the small sample size, and with 2 patients having bilateral FAIS, the data could be skewed. However, the patients were selected prospectively from a single surgeon at a high-volume hip preservation center and represent FAIS patients most commonly presenting for care. Although the study size was limited to 12 hips, the a priori power analysis determined that 9 hips would provide 80% power to detect a 25% change in peak joint moment. Ultimately, larger group sizes are required to draw clinical conclusions about postoperative biomechanics after hip arthroscopy. Biomechanical analysis was also limited to the hip joint, whereas a more comprehensive gait assessment may also include analysis of knee and ankle in the ipsilateral limb. Furthermore, biomechanical evaluation of normal walking gait alone may not be reflective of patient activity demand. Future studies may consider biomechanical analysis during more rigorous activities including running, jumping, cycling, and movements requiring larger range of motion.

## Conclusion

Gait analysis of FAIS patients treated with hip arthroscopy demonstrated small but significant changes in postoperative hip kinetics and kinematics. In particular, postoperative patients demonstrated increased peak hip abduction angle and decreased peak hip extension moments. These biomechanical changes, however, did not correlate with the large, clinically significant improvements in patient-reported outcomes at 1 year after surgery.
